# Supplementary material for: The Systems Biology Research Tool: evolvable open-source software
Source: BMC Syst Biol. 2008 Jun 29;2:55. doi: 10.1186/1752-0509-2-55 (PMC2446383; doi:10.1186/1752-0509-2-55)
Supplement: Additional file 1 — SBRT Archive. An archive of the current version of the Systems Biology Research Tool. [file 1752-0509-2-55-S1.zip › sbrt-1.4.0/doc/developers_guide/api/sbrt/shell/io/fba/RxnNamExprSetInputFile.html]

RxnNamExprSetInputFile


|  |  |  |
| --- | --- | --- |
| **Overview** \| **Package** \| **Class** \| **Use** \| **Tree** \| **Deprecated** \| **Index** \| **Help** | |  |
| **PREV CLASS**   **NEXT CLASS** | **FRAMES**    **NO FRAMES**     **All Classes** |  |
| SUMMARY: NESTED \| FIELD \| CONSTR \| METHOD | DETAIL: FIELD \| CONSTR \| METHOD |  |


---


## sbrt.shell.io.fba Class RxnNamExprSetInputFile<E extends MathExpr<S>,S>

```
java.lang.Object
  sbrt.shell.io.TextInputFile<E>
      sbrt.shell.io.fba.RxnNamExprSetInputFile<E,S>
```

**Type Parameters:**: `E` - the mathematical expression type.: `S` - the variable type.

**All Implemented Interfaces:**: java.lang.Iterable<E>, InputFile<E>, SbrtFile

---

``` public class RxnNamExprSetInputFile<E extends MathExpr<S>,S> extends TextInputFile<E> ```

This class is used to read files containing a set of reaction name
expressions. A single expression occurs on each line of the file. If the file
contains identical expressions, a `FormatException` is thrown.

**Author:**
:   This class was written and documented by
    Jeremiah Wright while in the Wagner lab.

---

**Constructor Summary**

`RxnNamExprSetInputFile(java.lang.String fileName,
RxnNameExprFormat<E,S> lineFormat)`
  
          Constructs a new reaction name expression set input file.


| **Method Summary** | |
| --- | --- |
| `UnmodifiableSet<E>` | `getRxnNameExprSet()`             Returns the set of reaction name expressions contained in this fle. |
| `java.util.Iterator<E>` | `iterator()`             Returns an iterator over the set of reaction name expressions contained in this file. |

**Methods inherited from class sbrt.shell.io.TextInputFile**

`close, finalize, getBasic, getBasic, getCanonicalName, getCommentChars, getFileFormat, getLineFormat, getOriginalName, isClosed, isValidFormat, iterator, lineIterator`

**Methods inherited from class java.lang.Object**

`clone, equals, getClass, hashCode, notify, notifyAll, toString, wait, wait, wait`

**Constructor Detail**

### RxnNamExprSetInputFile

```
public RxnNamExprSetInputFile(java.lang.String fileName,
                              RxnNameExprFormat<E,S> lineFormat)
```

:   Constructs a new reaction name expression set input file.

    **Parameters:**: `fileName` - the name of the file to be read.: `lineFormat` - the format used to parse data strings.


**Method Detail**

### getRxnNameExprSet

```
public UnmodifiableSet<E> getRxnNameExprSet()
```

:   Returns the set of reaction name expressions contained in this fle.

    :   **Returns:**: the set of reaction name expressions contained in this fle.

---


### iterator

```
public java.util.Iterator<E> iterator()
```

:   Returns an iterator over the set of reaction name expressions contained
    in this file.

    :   **Specified by:**: `iterator` in interface `java.lang.Iterable<E extends MathExpr<S>>` **Overrides:**: `iterator` in class `TextInputFile<E extends MathExpr<S>>`
    :   **Returns:**: an iterator over the set of reaction name expressions contained in this file.


---


|  |  |  |
| --- | --- | --- |
| **Overview** \| **Package** \| **Class** \| **Use** \| **Tree** \| **Deprecated** \| **Index** \| **Help** | |  |
| **PREV CLASS**   **NEXT CLASS** | **FRAMES**    **NO FRAMES**     **All Classes** |  |
| SUMMARY: NESTED \| FIELD \| CONSTR \| METHOD | DETAIL: FIELD \| CONSTR \| METHOD |  |


---
